# Responses of the microbial community and the production of extracellular polymeric substances to sulfamethazine shocks in a novel two-stage biological contact oxidation system

**DOI:** 10.3389/fmicb.2023.1240435

**Published:** 2023-08-29

**Authors:** Jia Zhou, Tian Chen, Jing Cui, Yan Chen, Shuai Zhao, Jian-Hang Qu, Zitong Wang, Jingshi Pan, Lixin Fan

**Affiliations:** ^1^School of Biological Engineering, Henan University of Technology, Zhengzhou, Henan, China; ^2^College of International Education, Henan University of Technology, Zhengzhou, Henan, China

**Keywords:** sulfadimethoxine, biological contact oxidation technology, microbial community, extracellular polymeric substances, antibiotic-resistant genes

## Abstract

**Introduction:**

The biological contact oxidation reactor is an effective technology for the treatment of antibiotic wastewater, but there has been little research investigating its performance on the sulfamethazine wastewater treatment.

**Methods:**

In this study, a novel two-stage biological contact oxidation reactor was used for the first time to explore the impact of sulfamethazine (SMZ) on the performance, microbial community, extracellular polymeric substances (EPS), and antibiotic-resistant genes (ARGs).

**Results:**

The chemical oxygen demand (COD) and ammonia nitrogen (${\text{NH}}_{4}^{+}$-N) removal efficiencies kept stable at 86.93% and 83.97% with 0.1–1 mg/L SMZ addition and were inhibited at 3 mg/L SMZ. The presence of SMZ could affect the production and chemical composition of EPS in the biofilm, especially for the pronounced increase in TB-PN yield in response against the threat of SMZ. Metagenomics sequencing demonstrated that SMZ could impact on the microbial community, a high abundance of *Candidatus_Promineofilum, unclassified_c__Anaerolineae*, and *unclassified_c__Betaproteobacteria* were positively correlated to SMZ, especially for *Candidatus_Promineofilum*.

**Discussion:**

*Candidatus_Promineofilum* not only had the ability of EPS secretion, but also was significantly associated with the primary SMZ resistance genes of *sul*1 and *sul*2, which developed resistance against SMZ pressure through the mechanism of targeted gene changes, further provided a useful and easy-implement technology for sulfamethazine wastewater treatment.

## Graphical Abstract

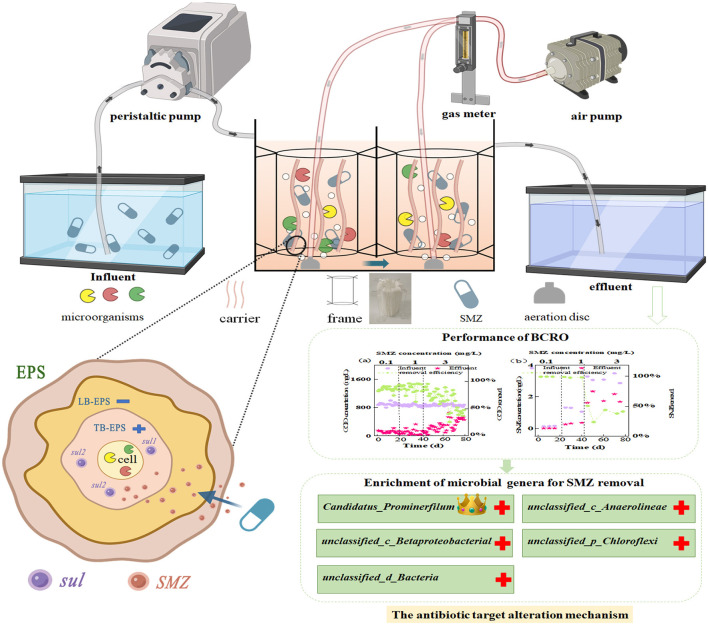

## 1. Introduction

Antibiotics are widely used due to their good antibacterial or bactericidal effects (Harada, 2018), which are widespread in sewage treatment plants, sewers, landfill leachate, river runoff, and waste discharge from livestock farms (Zhang et al., 2022b). The widespread detection of antibiotic residues in various environments will accelerate the spread of antibiotic-resistant bacteria (ARB) and ARGs, further altering the microbial community and threatening ecological safety and human health (Bai et al., 2016; Ben et al., 2018). Among the various types of antibiotics, sulfonamides are detected with substantial frequency in the environment due to their broad antibacterial activity and high potency at a low cost (Acosta-Rangel et al., 2019). In particular, the typical short-acting sulfamethazine (SMZ) was considered the most emerging contaminant among other sulfur groups containing medicine, which was most frequently used in inhibiting the growth of gram-positive bacteria due to its two ionizable functional groups: the aniline amine and the amide moieties (Gao and Pedersen, 2005; Du et al., 2018). The levels of SMZ in the environment ranged from ng/L to μg/L (Li et al., 2019). Therefore, it is urgent to develop efficient approaches for removing SMZ from the environment (Fu et al., 2020).

In recent years, catalytic ozonation (Bai et al., 2016), Fenton (Zhuang and Wang, 2021), and photodegradation (Ouyang et al., 2020) have been used for SMZ wastewater treatment. However, it is limited due to large secondary pollution and economic costs. Our previous studies have confirmed that the biological contact oxidation reactor (BCOR) is a promising technology for the treatment of chloramphenicol wastewater due to its structural setup (Zhou et al., 2022), which is beneficial to the separation of microorganisms and diminishes interspecific competition, further removing the antibiotics step by step upon direct contact with wastewater. In this process, the microbial community in activated sludge played a crucial role in improving the performance of biotechnology in removing antibiotics from wastewater, although the antibiotics may cause stress to the microbes or even alter their community structure (Mulla et al., 2021). Furthermore, a shift in the associated microbial communities may disrupt the stability and performance of the reactor (Strous et al., 2006).

Previous studies have suggested that microbial resistance to SMZ may be related to the secretion of EPS (Zhang et al., 2019). EPS is not only an important reservoir for antibiotics but also contains active enzymes that can remove antibiotics, which was a response to the antibiotic stress to protect themselves and adhere to each other. Meanwhile, EPS also influenced the physicochemical and biological functions of activated sludge, especially for its main structures of tightly bound EPS (TB-EPS) and loosely bound EPS (LB-EPS) (Yu et al., 2023). As the key component of EPS (Wu et al., 2022), PN can transport small molecules and further facilitate the adsorption of antibiotics from wastewater through the amino and carboxyl groups (Song et al., 2023). These findings suggest that EPS might play an important role in the removal of SAs. However, less attention has been focused on investigating the effect of EPS on SMZ removal, especially the evolvement action roles of microorganisms and EPS against SMZ shocks during the SMZ wastewater treatment using BCOR, which is currently unclear.

In this study, a novel two-stage biological contact oxidation reactor was constructed to (1) investigate the long-term impacts of increasing SMZ stress on the performance; (2) explore the production and chemical composition of EPS produced by the activated sludge against SMZ shocks; and (3) reveal the shift of the microbial community and the abundance of ARGs with the gradient concentrations of SMZ by the metagenomics sequencing technology. The results highlight the crucial roles of microbial EPS in responding to different concentrations of SMZ shocks during biological wastewater treatment.

## 2. Materials and methods

### 2.1. Experimental setup

**Figure 1:**
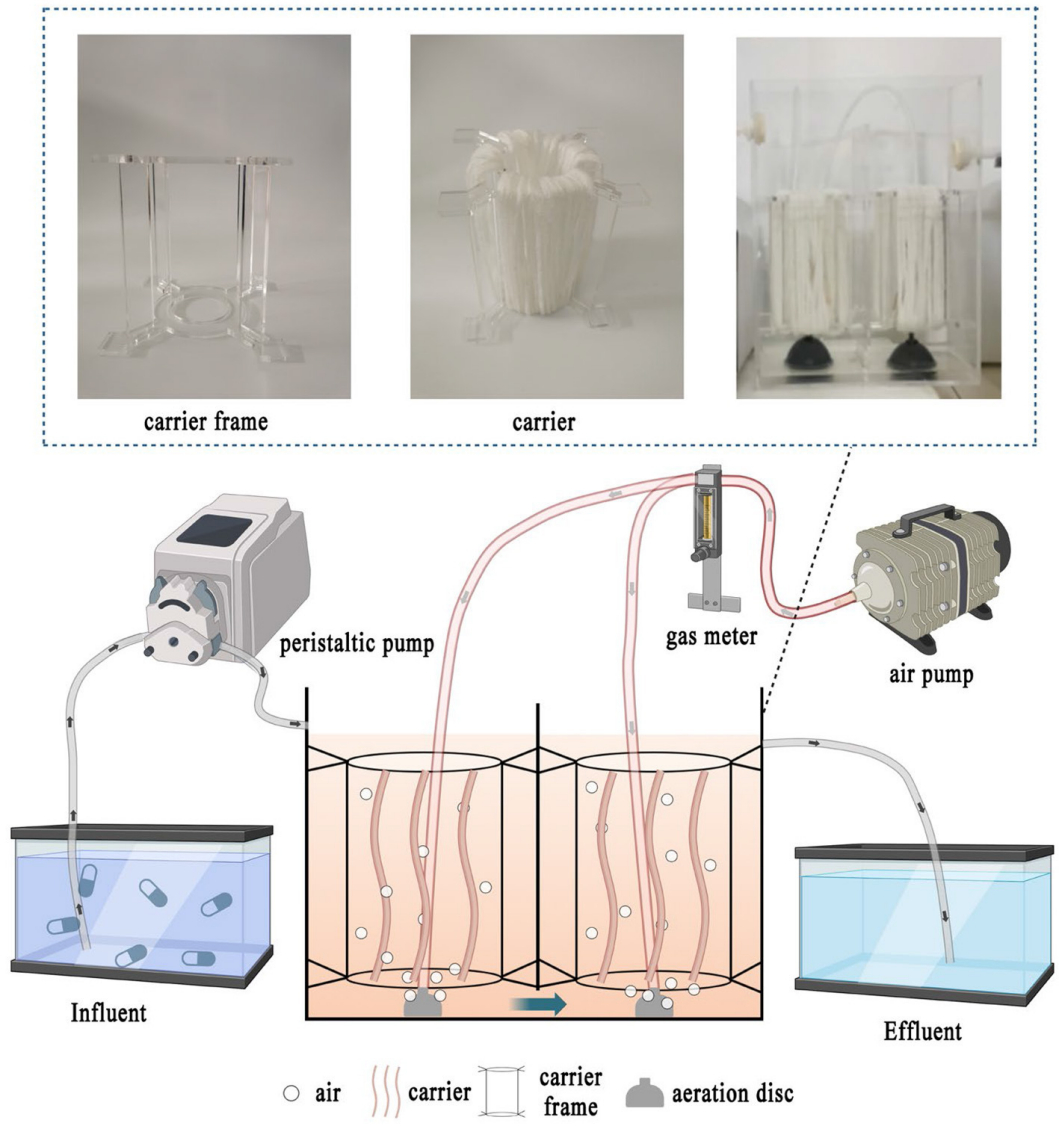
Two-stage biological contact oxidation reactor process flow diagram.

A two-stage biological contact oxidation reactor with an effective volume of ~6 L was set up in Figure 1, which is composed of two identical rectangular chambers (125 × 125 × 200 mm). The influent was pumped (BT101s, Lead Fluid, China) into the inlet and flowed to the outlet downward and upwardly through the overflow ports on both sides of the bottom in the middle baffle of the two chambers. The microporous aeration disc (LZB-3WB, Xiangjin, China) was fixed at the bottom of each chamber with dissolved oxygen (DO) of 3–4 mg/L. The acrylic zigzag circle frame with top and bottom inner diameters of 50 and 60 mm was suspended and fixed at 50–100 mm above the aeration disc in each chamber through its four serrations. Furthermore, the mixed polyethylene carrier (Teijin, Japan), which has a compact and porous fiber structure with a filling capacity yield of 3.5 g/L carriers, was wrapped around the zigzag circle frame to serve as a filter that absorbed the activated sludge and formed an efficient biofilm.

### 2.2. Biofilm culturing

Seed sludge was gathered from the secondary sedimentation tank of the Wulongkou Municipal Wastewater Treatment Plant (MWTP). The original mixed liquor-suspended solids (MLSS) of the two chambers were maintained at ~4.0 g/L. After aerating for 3 days, the sludge was almost adsorbed onto the carrier surface, and the liquid was clarified. Afterward, the microorganisms were inhabited at a high density in the activated sludge, which is beneficial to forming a biofilm and realizing the selective distribution and natural differentiation of microorganisms in space and function through synergy and antagonism.

### 2.3. Wastewater components

Initially, 0.05 g of standard SMZ was dissolved in a small amount of 50 mL of acetonitrile (HPLC grade) and then stored in amber-colored vials at −20°C. After the completion of biofilm incubation, the synthetic SMZ wastewater was prepared based on the previous study (Shi et al., 2018; Wan et al., 2018), with the main composition of CH_3_COONa 1.15 g/L, NH_4_Cl 0.34 g/L, K_2_HPO_4_ 0.10 g/L, and trace elements of 1 mL/L (1.50 g/L FeCl_3_·6H_2_O, 0.15 g/L H_3_BO_3_, 0.03 g/L CuSO_4_·5H_2_O, 0.12 g/L MnCl_4_·H_2_O, 0.06 g/L NaMoO_4_·2H_2_O, 0.12 g/L ZnSO_4_·7H_2_O, 0.15 g/L CoCl_2_·6H_2_O). It was pumped into the reactor continuously at room temperature (25–30°C) for 80 days. The flow rate of the peristaltic pump was 4.25 mL/min to maintain the hydraulic retention time (HRT) of 24 h. The operation process could be generally divided into three phases based on SMZ shocks in the influent, which gradually increased from 0.10 to 1 mg/L and 3 mg/L, according to previous studies (Yang et al., 2015; Li et al., 2019).

### 2.4. Analysis of a wastewater sample

The influent and effluent samples were collected daily and centrifuged at 10,000 rpm for 10 min, and the supernatant was taken to determine COD, ${\text{NH}}_{4}^{+}$-N, and ${\text{PO}}_{4}^{3-}$ levels as previously described (Zhou et al., 2022). The DO and pH values were measured using a DO meter (YSI 550A, USA) and a pH meter (HQ30d, HACH, America). The supernatant samples were filtrated using a 0.22-μm filter membrane (PTFE) for SMZ quantification using an analytical liquid chromatograph (LC-2030C, Shimadzu, Japan), equipped with the SB-C18 chromatographic column (Agilent ZORBAX, 150 × 4.6 mm, 5 μm) at a detection wavelength of 270 nm, an injection volume of 20 μL, and a flow rate of 1 mL/min. A combination of two mobile stages was programmed with pure water (A) and acetonitrile (B), and the mobile stage elution gradient was 0–2 min 60% A, 2–4 min 70% A, and 4–6 min 75% A.

### 2.5. EPS extraction and component analysis

The seed sludge (CK) biofilm samples obtained from the carrier in the reactor at the end of stages 2 (Z1) and 3 (Z3) were used to extract the EPS using thermal extraction. First, 25 mL of the sludge mixture was centrifuged at 4,000 r/min for 5 min at 4°C to remove the supernatant. Then, the sludge was replenished to its original volume with PBS buffer (NaCl 8.5%, Na_2_HPO_4_ 2.2%, NaH_2_PO_4_ 0.4%, distilled water 100 mL, pH 7), heated to 70°C, mixed well, and centrifuged at 4,000 r/min for 10 min at 4°C to obtain the supernatant (LB-EPS). The supernatant was then supplemented with PBS buffer to its original volume and heated in a water bath at 70°C for 30 min. Then, it was centrifuged at 4,000 r/min for 15 min at 4°C to obtain the supernatant (TB-EPS). Afterward, solutions of LB-EPS and TB-EPS were filtered through a 0.45-μm membrane.

The protein (PN) and polysaccharide (PS) concentrations were determined using the Bradford method and the phenol-sulfuric acid method (Peng et al., 2022). The organic compositions and fluorescence characteristics in EPS were determined using three-dimensional excitation and emission matrix fluorescence (3D-EEM). The excitation wavelength (Ex) was scanned from 200 to 400 nm, and the emission wavelength (Em) was scanned from 300 to 500 nm, both in 5 nm increments (Zhang et al., 2022a).

### 2.6. Metagenomics sequencing

The seed sludge (CK) and the biofilm sludge samples collected at the end of stages 2 (Z1) and 3 (Z3) were used for metagenomics analysis on an Illumina HiSeq 4000 platform (Majorbio, China). The raw reads were used to generate clean reads using fastp (Chen et al., 2018) on the Majorbio Cloud Platform (cloud.majorbio.com). A non-redundant gene catalog was constructed using CD-HIT (Fu et al., 2012). The species abundance was calculated from the sum of gene abundances corresponding to the species. Representative sequences from the non-redundant gene catalog were annotated by Blastp based on the NCBI NR database, using DIAMOND for taxonomic annotation with an e-value cutoff of 1e^−5^. Antibiotic resistance gene annotation was conducted using DIAMOND (Buchfink et al., 2015) against the CARD database with an e-value cutoff of 1e^−5^. Network analysis was used to explore the potential correlations between bacteria and ARGs based on Spearman's correlation coefficient > 0.6 (*P* < 0.05). The data were deposited into the NCBI Sequence Read Archive (SRA) database under accession number PRJNA838398.

## 3. Results

### 3.1. Reactor performance

**Figure 2:**
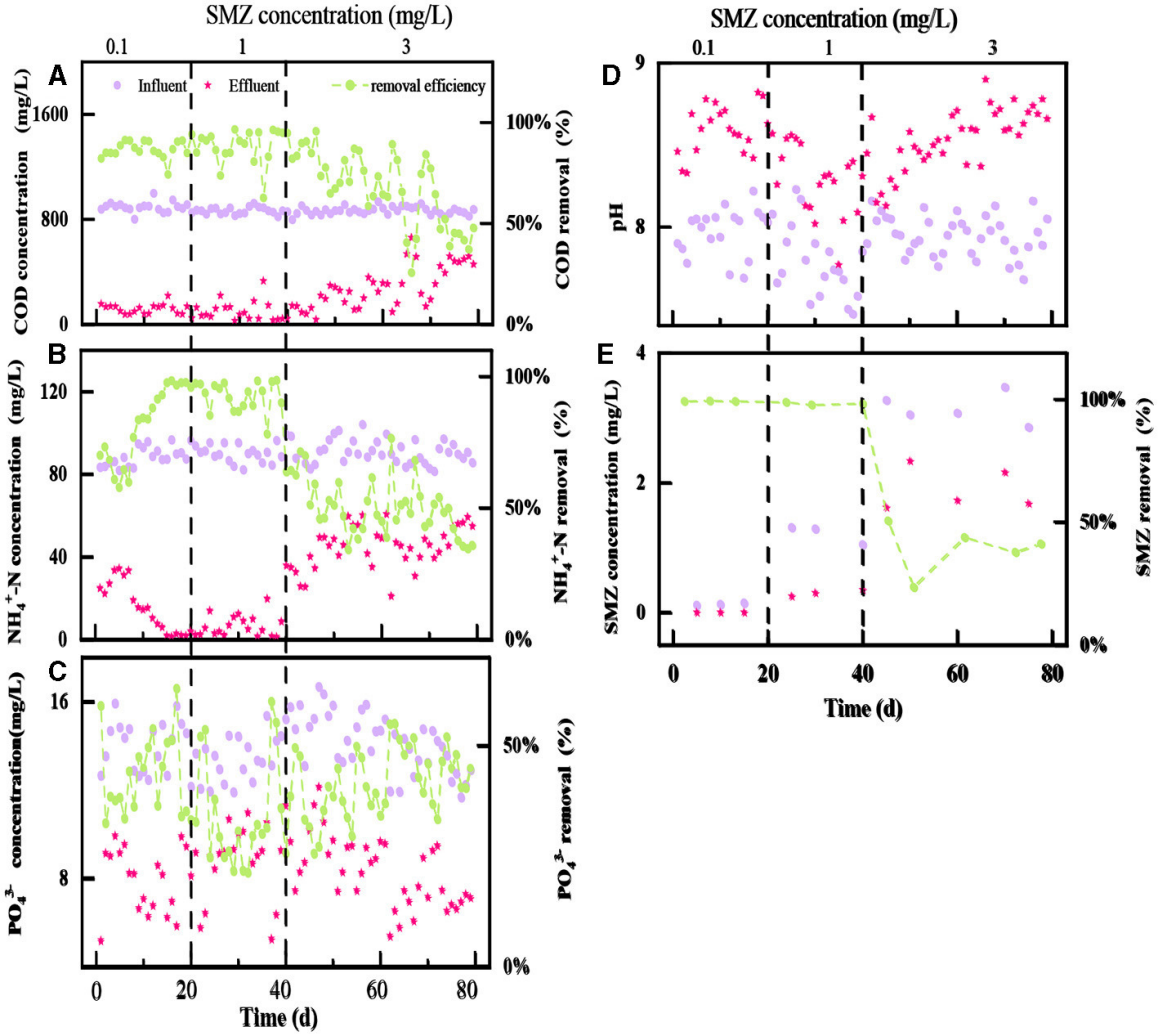
The variations of COD **(A)**, ${\text{NH}}_{4}^{+}$-N **(B)**, ${\text{PO}}_{4}^{3-}$ **(C)**, pH **(D)**, and SMZ **(E)** concentrations in influent and effluent at different stages.

The performance of the two-stage biological contact oxidation reactor for the treatment of SMZ wastewater is shown in Figure 2. The SMZ shocks in stages 1–2 barely influenced the performance of BCOR in COD removal (Figure 2A), and the average COD removal efficiency was 86.93 and 88.04%, respectively. However, the obvious fluctuation of COD removal efficiency appeared in stage 3, and it reduced to 66.39%, mainly attributed to the increasing SMZ stress. In addition, NH4+-N removal efficiency kept relatively stable at 83.87–90.03% when the SMZ concentration increased from 0.10 to 1 mg/L (Figure 2B), indicating that the nitrifying bacteria still had strong activity even under the increasing SMZ pressure. However, the NH4+-N removal efficiency illustrated a decreasing tendency when the SMZ concentration in the influent increased to 3 mg/L. The high SMZ concentration (3 mg/L) in the present research could impact the removal of organic matter and the process of ammonia oxidization.

Moreover, there was no significant difference in the effect of different SMZ concentrations on ${\text{PO}}_{4}^{3-}$ removal efficiency (Figure 2C), which always fluctuated around 42.12%. The pH of the effluent is higher than that of the influent (Figure 2D). It has been reported that pH affects phosphorus removal by the microbial community. With effluent pH exceeding 8, phosphorus precipitation tends to form in the system (Wang et al., 2022), affecting phosphorus utilization by microorganisms and reducing phosphorus removal efficiency. Overall, the two-stage biological contact oxidation reactor had a limited effect on the removal of ${\text{PO}}_{4}^{3-}$ during the SMZ wastewater treatment.

Afterward, SMZ was undetectable in the effluents during stage 1 (Figure 2E), indicating that activated sludge completely adsorbed or degraded the dosed SMZ. Afterward, the SMZ removal efficiency was above 90% in stage 2. In stage 3, the average removal efficiency of SMZ decreased to 39.36% with 3 mg/L SMZ added to the influent. The gradient concentrations of SMZ resulted in a significant reduction in the performance of the two-stage biological contact oxidation reactor during SMZ wastewater treatment.

### 3.2. Response of EPS production to SMZ shocks

**Figure 3:**
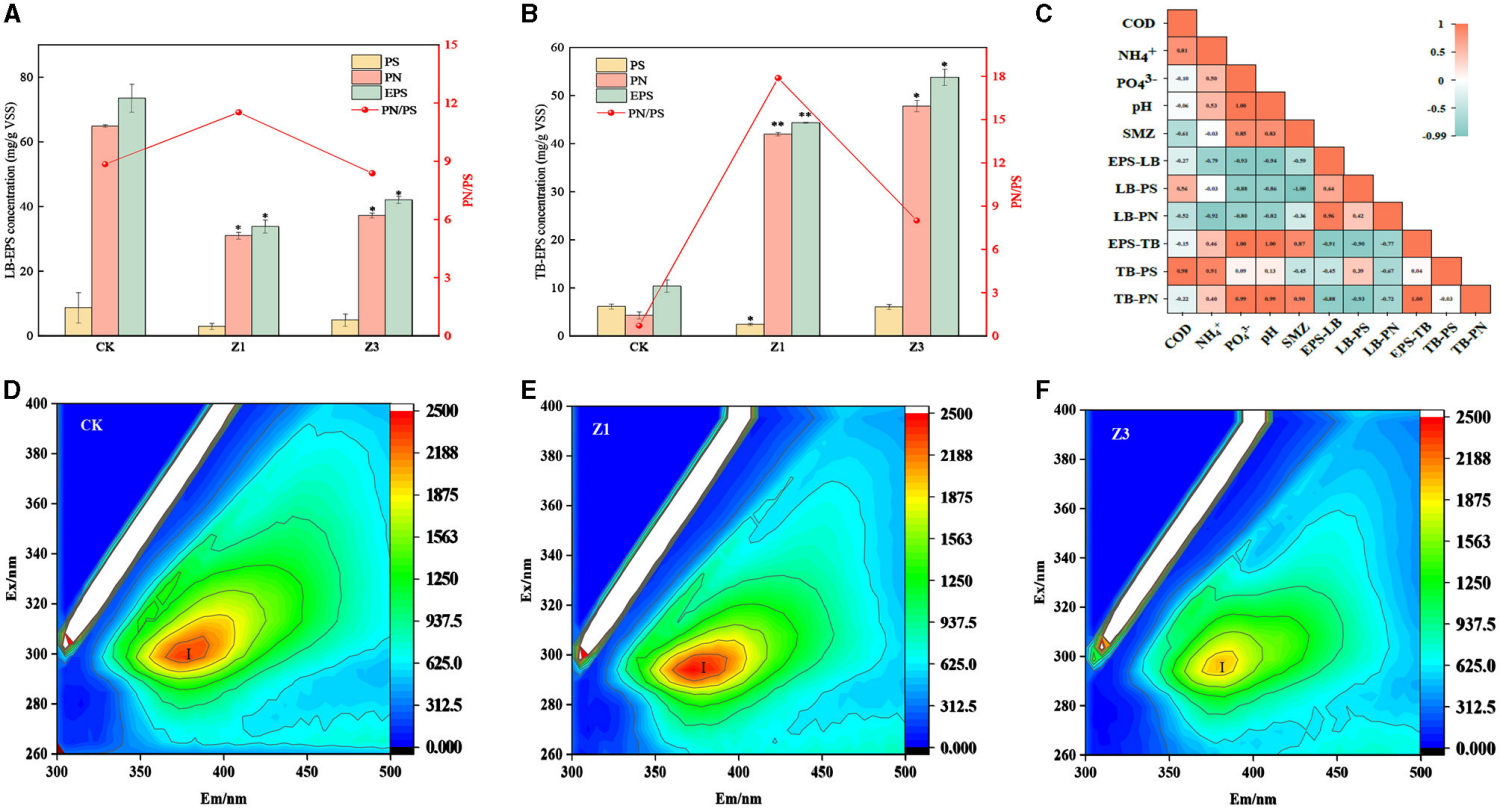
Variations of LB-EPS **(A)** and TB-EPS **(B)** concentration at different stages (CK: seeded sludge; Z1 and Z3, indicated the sludge collected at the end of stage 2, 3); correlation analysis of extracellular polymers with environmental factors **(C)**; excitation-emission matrix fluorescence spectra for EPS in CK **(D)**, Z1**(E)**, and Z3 **(F)**.

Biofilms are complex structures composed of microorganisms, and EPS, as the main component of biofilms, is a key factor affecting the performance of reactors (Xiong et al., 2020). EPS content is strongly influenced by SMZ in Figure 3. The addition of SMZ to the influent inhibited the secretion of LB-EPS (Figure 3A), which was reduced from 73.53 mg/(g VSS) in CK to 42.04 mg/(g VSS) in Z3. On the contrary, the production of TB-EPS substantially increased after each SMZ shock to 44.33 mg/(g VSS) in Z1 and 53.79 mg/(g VSS) in Z3, respectively (Figure 3B), which was significantly higher than the 10.34 mg/(g VSS) in CK. It has been reported that EPS can resist antibiotic stress by the adsorption of antibiotics through the hydrophobic region and functional groups of PN (Fan et al., 2021). The pronounced increase in PN yield mainly contributed to the elevated TB-EPS production, reaching 41.97 mg/(g VSS) and 47.79 mg/(g VSS) in Z1 and Z3, respectively. Its production was promoted to protect microbial cells against SMZ shocks. In particular, SMZ is positively correlated with TB-EPS and TB-PN content and negatively correlated with LB-EPS content, while TB-EPS is also positively correlated with PN content (Figure 3).

The chemical composition of EPS was further investigated at different SMZ concentrations by the 3D-EEM fluorescence spectra in Figures 3D–F. The I-peak (250 nm < Ex < 400 nm, 200 nm < Em < 380 nm) was associated with aromatic protein-like substances, according to Zhu et al. (2022). The I-peak appeared at all SMZ concentrations, while its intensity significantly decreased with increasing SMZ concentrations. Compared to 0 mg/L SMZ, the location of the I-peak in TB-EPS was shifted by 20/20 nm and 25/25 nm along the excitation/emission scale at 1 and 3 mg/L SMZ, respectively. Additionally, the fluorescence shift further illustrated that SMZ could affect the chemical composition of EPS.

### 3.3. Effect of SMZ on the bacterial community

**Figure 4:**
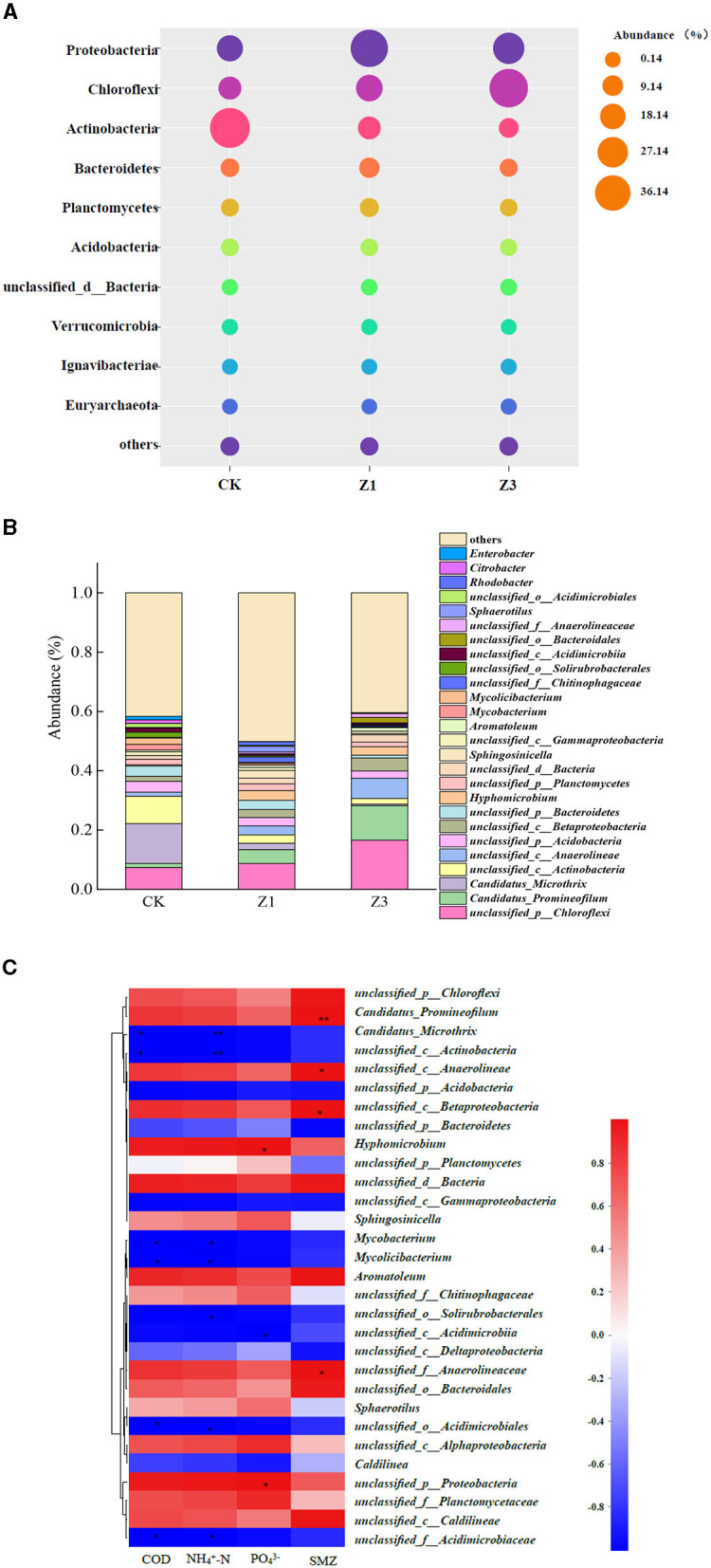
Phylum-level classification of bacterial communities (the relative abundance of <1% was defined as “others”) **(A)**; percentage of bacterial community abundance on genus level (the relative abundance of <1% was defined as “others”) **(B)**; a correlation heatmap of the top 30 genera related to environmental factors **(C)**. ^*^Represents *P* less than 0.05, ^**^represents *P* less than 0.01.

The impact of SMZ pressure on the microbial composition and distribution was investigated by the metagenomics sequencing technology in Figure 4. The bacterial community composition and abundance differed significantly with an increase in SMZ concentration, and the flora with poor environmental adaptability were gradually eliminated. At the phylum level, the abundance of Chloroflexi increased from 19.25% (CK) to 20.12% (Z1) and 41.75% (Z3) with an increase in SMZ concentration (Figure 4A). Moreover, the abundance of Proteobacteria reached 39.54% in Z1, while it decreased to 28.62% in Z3. In contrast, the abundance of Actinobacteria had an obvious decrease from 44.22% (CK) to 8% (Z3) under the selection pressure of SMZ in the environment.

At the genus level (Figure 4B), *Candidatus_Microthrix* and *unclassified_c__Actinobacteria* were the most prevalent genera in CK, accounting for 22.6% of all observed sequences. However, their abundances showed a downward trend and reduced to 0.5 and 1.9% in Z3, respectively, with the increased SMZ concentration, probably because the SMZ concentrations exceeded these bacteria' tolerance levels. In contrast, the abundances of *unclassified_d__Bacteria, unclassified_c__Betaproteobacteria, unclassified_c__Anaerolineae, Candidatus_Promineofilum*, and *unclassified_p__Chloroflexi* increased with the increased SMZ concentration, especially for *Candidatus_Promineofilum*, whose abundance increased from 1.4% in CK to 11.6% in Z3, indicating higher levels of adaptation for SMZ. Furthermore, the SMZ was significantly correlated with *Candidatus_Promineofilum* (*P* < 0.05), followed by *unclassified_c__Anaerolineae, unclassified_c__Betaproteobacteria*, and *unclassified_f__Anaerolineaceae* (*P* < 0.05) (Figure 4C). Meanwhile, the relative abundances of genera *Candidatus_Promineofilum, unclassified_c__Betaproteobacteria*, and *unclassified_f__Anaerolineaceae* exhibited an increasing trend in the SMZ wastewater treatment system (Figure 4B), further demonstrating their powerful tolerance to SMZ.

### 3.4. Co-occurrence associations between the microbial community and ARGs

**Figure 5:**
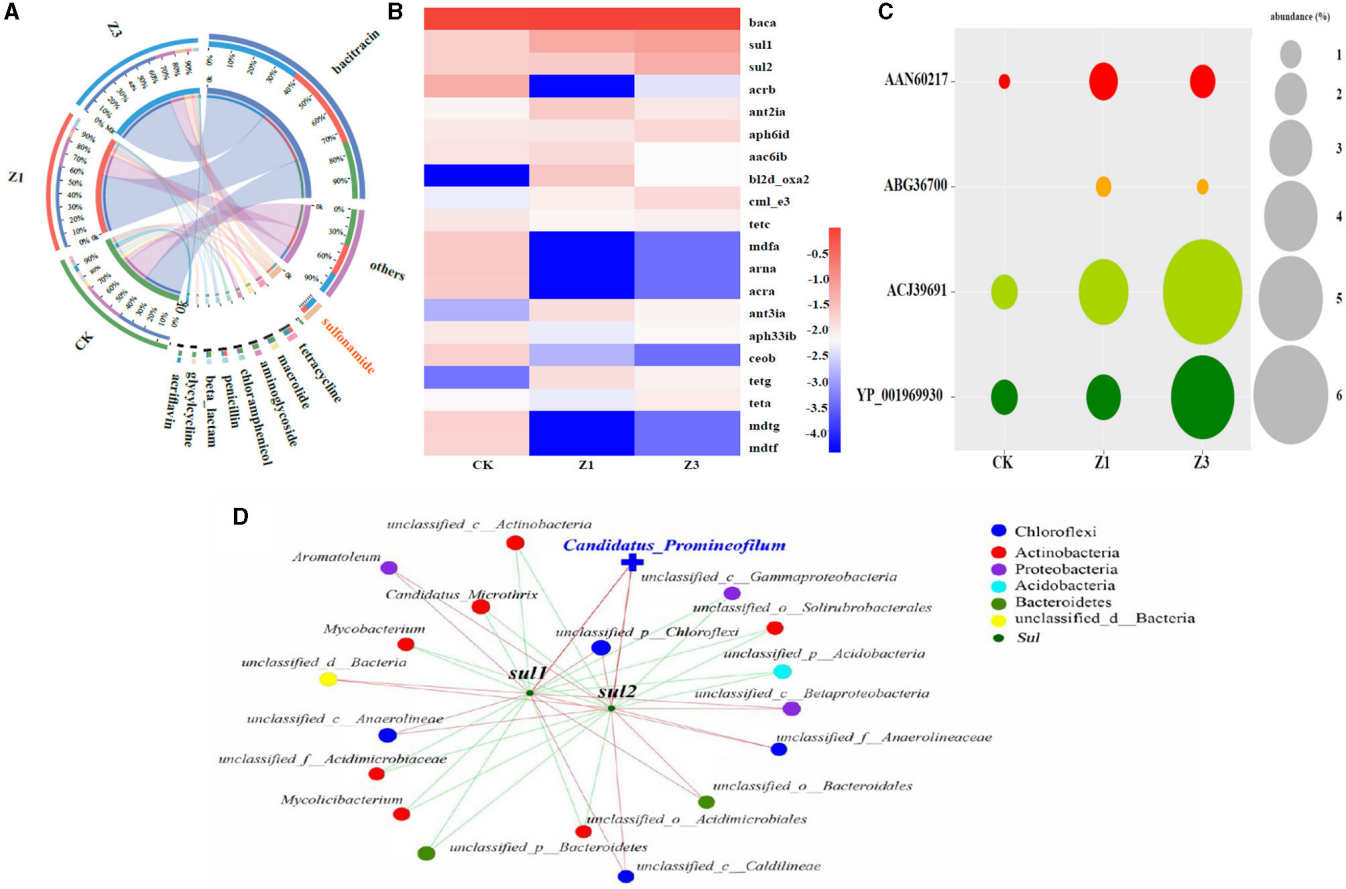
Circos analysis between the biofilm samples and antibiotic type (others: abundance < 3%) **(A)**; heatmap analysis of the top 20 ARGs types in each sample **(B)**; the abundance of ARGs associated with SMZ **(C)**; network analysis revealing the co-occurrence patterns among the ARGs types associated with SMZ and the top 20 genera **(D)**.

The presence of SMZ not only affects the microbial community but also leads to the development, migration, and transformation of diverse ARGs. Therefore, the correlation between the abundance of ARGs and the microbial community has been further explored in Figure 5. During the SMZ wastewater treatment system, the types of antibiotics with higher abundance in the sludge samples from CK, Z1, and Z3 were bacteriocins and sulfonamides. The abundance of sulfonamide antibiotics increased substantially from 12% (CK) to 31% (Z1) and 56% (Z3), respectively (Figure 5A). This phenomenon indicated that the SMZ concentration in the influent serves as a crucial factor for enriching its corresponding sulfonamide antibiotics. Among these ARGs (Figure 5B), *bacA, sul*1, and *sul*2 were the top three dominant genes across the three samples. *sul*1 and *sul*2 are the sulfonamide resistance genes, and their abundances increased with the increasing SMZ concentration, which enhanced the propagation and transformation of ARGs among bacteria. Afterward, AAN60217, ABG36700, and ACJ39691 associated with *sul*1, and YP_001969930 associated with *sul*2 also exhibited an increasing trend during the SMZ wastewater treatment (Figure 5C), further revealing that the selection pressure imposed by increased SMZ promoted the occurrence of these associated bacteria. The co-occurrence pattern of *sul*1, *sul*2, and microbial communities revealed that the 19 bacterial genera were speculated as possible hosts (Figure 5D). The microorganisms positively associated with *sul*1 and *sul*2 were mainly attributed to Chloroflexi and Proteobacteria, especially *Candidatus_Promineofilum*. It was not only strongly correlated with *sul*1 and *sul*2, but it was also the most enriched genus associated with the removal of SMZ in this system.

### 3.5. Antibiotic resistance mechanism

**Figure 6:**
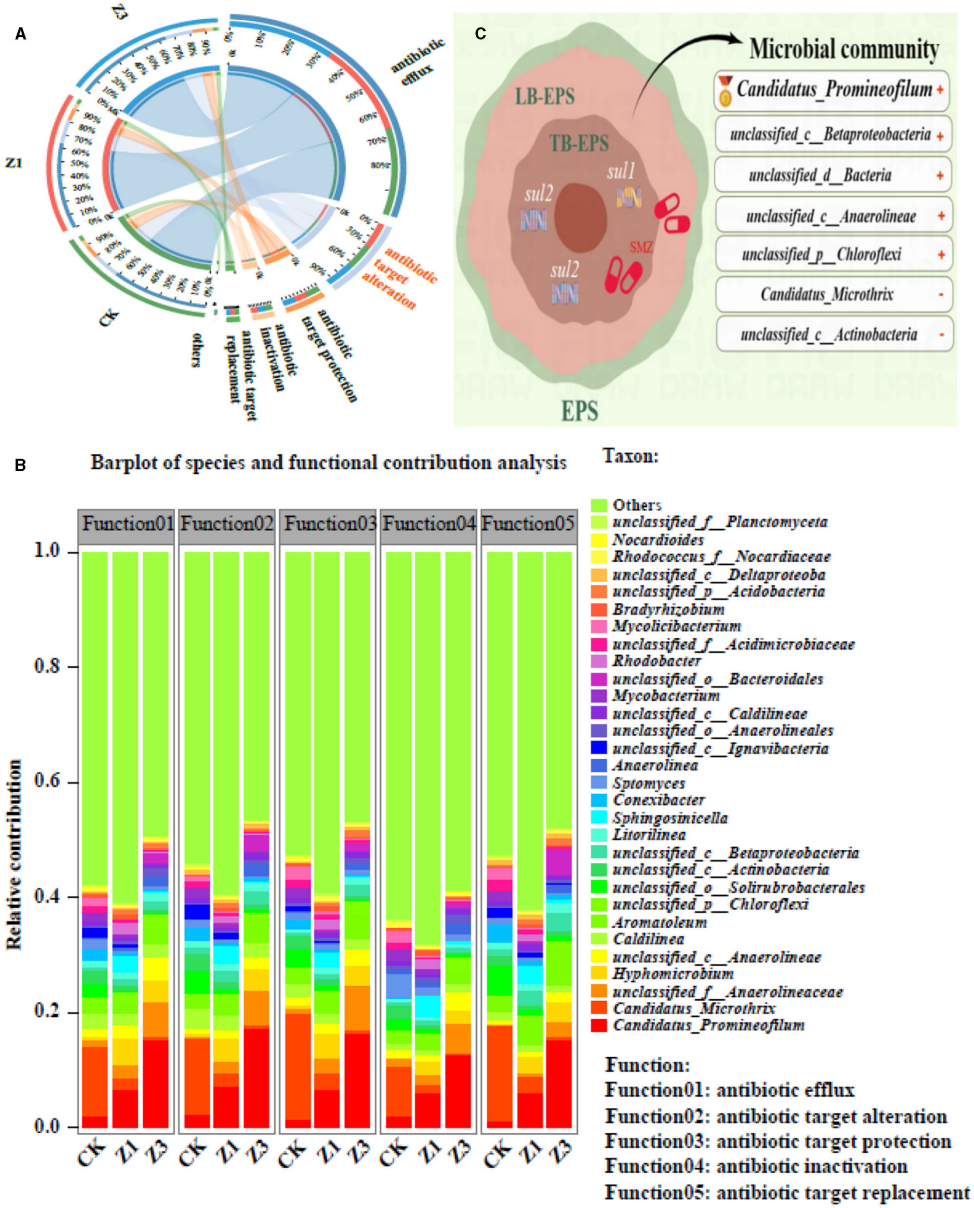
Circos analysis between the biofilm samples and resistance mechanism (others: abundance < 1%) **(A)**; relative contributions for bacterial genera (top 30 genera) to resistance mechanisms **(B)**; the mechanisms of tolerance and removal of SMZ **(C)**.

Antibiotics exert selective pressure on microbial communities and promote the emergence of ARGs. Sulfonamide antibiotics were dissipated and driven by microbes mainly through the initial ipso-hydroxylation (Chen et al., 2022). To analyze the factors that contribute to the mechanism of resistance of biota to SMZ, it is crucial to understand the mechanism of action of antibiotics. During the SMZ wastewater treatment system, the antibiotic target alteration was persistently prevalent in different SMZ concentrations (Figure 6A), which was considered a primary resistance mechanism for bacterial resistance to SMZ, among which the relative contribution of *Candidatus_Promineofilum, unclassified_f__Anaerolineaceae, Hyphomicrobium, unclassified_c__Anaerolineae*, and *unclassified_p__Chloroflexi* to this resistance mechanism gradually increased with the increased SMZ concentration (Figure 6B), indicating that the members of these genera could resist the adverse environment of SMZ pressure through antibiotic target alteration mechanisms (Figure 6C).

## 4. Discussion

The two-stage biological contact oxidation reactor exhibited excellent performance in removing low concentrations (0.1–1 mg/L) of SMZ with a removal rate higher than 90% (Figure 2E), which was higher than the 56.0% removal rate reported by Yang et al. (2015) for the treatment of 1 mg/L SMZ using the lab-scale sequencing batch reactor. However, the SMZ, COD, and ${\text{NH}}_{4}^{+}$-N removal efficiencies all had a decreasing trend when the SMZ concentration in the influent increased to 3 mg/L. This phenomenon was similar to Yang et al. (2015) in that SMZ was not completely removed by the acclimatized activated sludge. However, it was lower than the 95.4% removal under 0.1–5 mg/L SMZ during the MBR system (Shi et al., 2018). There are huge differences in the biodegradation of SMZ, which might be due to the differences in study conditions (including activated sludge) and also from place to place. The addition of the antibiotic SMZ affects the structure of a bacterial community in this system. The biofilm is subjected to greater environmental stress, and the species abundance is affected by the increase in time and SMZ concentration (Ciofu et al., 2022).

The EPS is essential for providing stability and adhesion of biofilms to the carrier in the reactor (Das, 2021). The TB-EPS contents kept an increasing tendency with an increase in SMZ concentration (Figure 3B), indicating that SMZ stimulated the microbial secretion of TB-EPS in the biofilm, especially given the pronounced increase in PN yield. PN plays a key role in the response to changes in environmental conditions. However, the variation trend in TB-EPS was opposite to that in LB-EPS, which may be because SMZ could migrate into the activated sludge by dynamically complexing with EPS. TB-EPS plays a central role in the protection of microbial cells, which can bind with antibiotics and secrete active enzymes for the removal of antibiotics, which then can alleviate cellular osmotic pressure imbalance (Sun et al., 2022). Furthermore, as the first barrier of microbial cells, EPS also contacts directly with SMZ in aqueous environments (Guo et al., 2020) and plays a key role in the biosorption of SMZ (Wang et al., 2018), further reducing it to undetectable levels in the effluent (Xu and Sheng, 2020). Consequently, microorganisms in the biofilm produced the EPS to form a network structure outside the cells, owing to the self-protection strategy against the toxicity of SMZ (Zhang et al., 2016).

The performance of BCOR in the treatment of SMZ wastewater is closely related to the key bacteria associated with SMZ biodegradation in activated sludge. Chloroflexi and Proteobacteria were enriched with an increase in SMZ concentration and became the dominant phylum (Figure 4A). Furthermore, Chloroflexi is involved in pathways for the complete hydrolytic or oxidative degradation of various recalcitrant organic matter, including aromatic compounds, polyaromatic hydrocarbons, polychlorobiphenyls, and organochlorine compounds (Liu et al., 2022). Proteobacteria play an important role in the degradation or transformation of sulfonamide compounds (Reis et al., 2018). Meanwhile, the abundance of *unclassified_d__Bacteria, unclassified_c__Betaproteobacteria, unclassified_c__Anaerolineae, Candidatus_Promineofilum*, and *unclassified_p__Chloroflexi* exhibited an increasing trend with the gradient concentrations of SMZ, especially for *Candidatus_Promineofilum* belonging to Chloroflexi. It was not only highly correlated with SMZ (Figure 4C) but also had the strongest correlation with the primary resistance genes to sulfonamides: *sul*1 and *sul*2 (Figure 5B), which may be their potential hosts. Previous studies have reported that *Candidatus_Promineofilum* is one of the most abundant populations in activated sludge (McIlroy et al., 2016), which contributed predominantly to aromatic degradation, quinoline degradation, and denitrification (Tian et al., 2023). Moreover, *Candidatus_Promineofilum* plays essential roles in EPS secretion, containing the exopolysaccharide biosynthesis genes (glmS and wbpA) and amino acid biosynthesis genes (ltaE and metH) (Dong et al., 2023). This provides an opportunity to protect and regulate itself by producing the EPS under the SMZ stress, and the increased levels of EPS further facilitate the SMZ adsorption.

Bacteria will adopt appropriate strategies to break the inhibitory effect of antibiotics according to the antibacterial mechanism of antibiotics, including mutation of the drug target (Garcia-Bustos et al., 2022), secretion of hydrolase (Pulingam et al., 2022), and excretion of antibiotics from cells through an efflux pump (Zhou et al., 2023). Previous studies have reported that bacterial resistance to sulfonamides is mainly associated with mutations in the gene encoding dihydrofolate synthetase (DHPs) in the chromosome, which reduces the affinity between DHPs and sulfonamides. Sul, as an alternative gene to DHPs (Aslam et al., 2012), has a much lower affinity for sulfanilamide and is the most common way to generate a sulfonamide resistance mechanism (Figure 6B). Therefore, in the microbial community, *unclassified_d__Bacteria, unclassified_c__Betaproteobacteria, unclassified_c__Anaerolineae, Candidatus_Promineofilum*, and *unclassified_p__Chloroflexi* in the SMZ-treated wastewater system mainly resist SMZ stress through targeted gene changes (Figure 6A).

## 5. Conclusions

The two-stage biological contact oxidation reactor displayed stable and efficient removal of SMZ (90%) at 0.1–1 mg/L SMZ, which was strongly correlated with the composition of EPS, especially for TB-PN played an important role in responding to the threat of SMZ. Moreover, SMZ has an obvious effect on the bacterial community. *Candidatus_Promineofilum*, with the ability to secrete EPS, was enriched mostly with the gradient SMZ concentrations, which accounted for 11.6% of the overall microbial community and became the predominant SMZ-degrading bacteria genus in BCOR biofilm. Furthermore, *Candidatus_Promineofilum* was not only highly correlated with SMZ but also had the strongest correlation with the primary sulfonamide resistance genes of *sul*1 and *sul*2. It could fight against SMZ stress through the mechanism of targeted gene changes.

## Data availability statement

The datasets presented in this study can be found in online repositories. The names of the repository/repositories and accession number(s) can be found in the article/supplementary material.

## Author contributions

TC: writing—original draft. JC: methodology and investigation. YC: methodology, resources, and supervision. SZ: project administration. J-HQ: visualization and writing—review and editing. ZW: conceptualization. JP: formal analysis. LF: supervision. All authors contributed to the article and approved the submitted version.
